# QUANTIFYING DIFFERENCES IN MITOCHONDRIAL RETICULUM OF HEALTHY OLD AND PERTURBED HUMAN MUSCLE USING AI SEGMENTATION

**DOI:** 10.1093/geroni/igae098.3697

**Published:** 2024-12-31

**Authors:** Lisa Hartnell, Alexander Maltsev, Luigi Ferrucci

**Affiliations:** National Institutes on Aging, Baltimore, Maryland, United States; National Institutes on Aging, Baltimore, Maryland, United States; National Institutes on Aging, Baltimore, Maryland, United States

## Abstract

Several studies have shown that mitochondrial health and function decline with aging and chronic disease having deleterious consequences on mobility and cognition. To better understand these changes, we examined the structure of skeletal muscle mitochondria in participants in the Genetic and Epigenetic Signatures of Translational Aging Laboratory Testing (GESTALT) study and in older individuals with peripheral arterial disease (PAD). Image analyses was performed on volumes of muscle generated by Focused Ion Beam (FIB)-Scanning Electron Microscopy (SEM). FIB-SEM yields isotropic volumes that allow analysis with deep learning aided segmentation at near membrane resolution. As previously reported using volumetric imaging, we found that mitochondrial reticulum is a highly organized continuum of individual mitochondria around myofibers found close to the location of energy utilization. To perform a quantitative analysis, we developed a deep learning segmentation workflow and applied the process to image volumes of gastrocnemius muscle ranging from 10-20 □m3 collected from healthy old (75-89y) participants in GESTALT, in frail individuals and in patients diagnosed with peripheral artery disease (PAD). We found substantial differences in the reticulum size, with fragmentation in frailty and PAD patients compared to healthy controls. We present our progress toward developing a measure of the breakdown of continuity of reticulum we termed “Fragmentation Index”. Future work aims to increase the number of individuals sampled and to apply the Fragmentation Index along with other measures of mitochondrial activity to evaluate the association of continuous mitochondria with healthy aged muscle vs muscle from frail individuals or individuals with PAD.

